# Treatment of biofilms in bacterial vaginosis by an amphoteric tenside pessary-clinical study and microbiota analysis

**DOI:** 10.1186/s40168-017-0326-y

**Published:** 2017-09-13

**Authors:** Cornelia Gottschick, Zhi-Luo Deng, Marius Vital, Clarissa Masur, Christoph Abels, Dietmar H. Pieper, Manfred Rohde, Werner Mendling, Irene Wagner-Döbler

**Affiliations:** 1 Research Group Microbial Communication, Helmholtz Centre for Infection Research, Inhoffenstr. 7, 38124 Braunschweig, Germany; 2 Research Group Microbial Interactions and Processes, Helmholtz Centre for Infection Research, Inhoffenstr. 7, 38124 Braunschweig, Germany; 3 Central Facility for Microscopy, Helmholtz Centre for Infection Research, Inhoffenstr. 7, 38124 Braunschweig, Germany; 4 Dr. August Wolff GmbH & Co. KG Arzneimittel, Sudbrackstrasse 56, 33611 Bielefeld, Germany; 5 German Center for Infections in Gynecology and Obstetrics, Wuppertal, Germany

**Keywords:** Bacterial vaginosis, Biofilms, Amphoteric tenside, Vaginal microbiome, Vaginal microbiota

## Abstract

**Background:**

Bacterial vaginosis (BV) is the most common vaginal syndrome among women in their reproductive years. It is associated with an increased risk of acquiring sexually transmitted infections and complications like preterm labor. BV is characterized by a high recurrence rate for which biofilms frequently found on vaginal epithelial cells may be a reason.

**Results:**

Here, we report a controlled randomized clinical trial that tested the safety and effectiveness of a newly developed pessary containing an amphoteric tenside (WO3191) to disrupt biofilms after metronidazole treatment of BV. Pessaries containing lactic acid were provided to the control group, and microbial community composition was determined via Illumina sequencing of the V1-V2 region of the 16S rRNA gene. The most common community state type (CST) in healthy women was characterized by *Lactobacillus crispatus.* In BV, diversity was high with communities dominated by either *Lactobacillus iners, Prevotella bivia*, *Sneathia amnii*, or *Prevotella amnii.* Women with BV and proven biofilms had an increased abundance of *Sneathia sanguinegens* and a decreased abundance of *Gardnerella vaginalis.* Following metronidazole treatment, clinical symptoms cleared, Nugent score shifted to Lactobacillus dominance, biofilms disappeared, and diversity (Shannon index) was reduced in most women. Most of the patients responding to therapy exhibited a *L. iners* CST. Treatment with WO 3191 reduced biofilms but did not prevent recurrence. Women with high diversity after antibiotic treatment were more likely to develop recurrence.

**Conclusions:**

Stabilizing the low diversity healthy flora by promoting growth of health-associated *Lactobacillus* sp. such as *L. crispatus* may be beneficial for long-term female health.

**Trial registration:**

ClinicalTrials.gov NCT02687789

**Electronic supplementary material:**

The online version of this article (doi:10.1186/s40168-017-0326-y) contains supplementary material, which is available to authorized users.

## Background

The Lactobacillus-dominated vaginal microbiota is protective against sexually transmitted infections [1]. Unlike most other body sites, it is characterized by a low diversity and uniform colonization by one of several species of *Lactobacillus* sp.. Five to eight community state types (CSTs) have been identified [2, 3]. The two most common ones are dominated by *L. crispatus* and *L. iners*, but CSTs dominated by *L. gasseri* and *L. jensenii*, and those dominated by *Gardnerella vaginalis* and other anaerobic bacteria have also been identified [4]. Together with a low pH (< 4.5) and a low Nugent score (0 to 3), these CSTs are characteristics of a healthy vagina, which is clinically characterized by white, normally smelling secretions [5]. Variation of the microbial community can be high within individuals and vary throughout the menstrual cycle, but the CST is usually stable in the long term [2].

Bacterial vaginosis (BV) is the most common vaginal syndrome in women of childbearing age, with a prevalence of 30% in Caucasian women [6] which are the majority of study participants analyzed here. This prevalence is based on the presence of abnormal microbial flora and can be asymptomatic [7]. BV is associated with a higher risk of acquiring sexually transmitted infections such as HIV, as well as miscarriage and preterm birth [4]. Furthermore, BV has a 60% rate of recurrence in the 12 months after treatment with the standard of care antibiotic metronidazole [8].

In BV, the community shifts to a highly diverse flora, concurrent with an increase in pH and a rise of Nugent score to > 6. The clinical expression of BV is gray-white, discharge with fishy odor due to elevated pH values. Taxa such as *Gardnerella*, *Atopobium*, *Prevotella*, *Bacteroides*, *Peptostreptococcus*, *Mobiluncus*, *Sneathia*, *Leptotrichia*, *Mycoplasma*, and BV-associated bacterium 1 (BVAB1) to BVAB3 of the order *Clostridiales* become abundant. Three CSTs dominated by *G. vaginalis*, *Lachnospiraceae*, and *Sneathia sanguinegens*, respectively, have been identified in BV [5]. Another characteristic of BV is the presence of a thick multispecies biofilm on vaginal epithelial cells [9]. Such cells are named “clue cells” because they serve as one out of the four diagnostic “Amsel” criteria [10]. Biofilms consist of adherent bacteria and the extracellular polymeric matrix they produce. This matrix inhibits biofilm elimination by the immune system and may prevent full destruction of the biofilm in bacterial vaginosis by antibiotics [11]. It has been shown that the biofilm is comprised mainly of *G. vaginalis* and *Atopobium vaginae* and persists on epithelial cells after treatment with antibiotics [9, 12]. The pathogenicity of *G. vaginalis* biofilms is demonstrated by their ability to adhere to epithelial cells even in the presence of *L. crispatus* and that they benefit from the addition of BV-related secondary colonizers [13, 14]. Furthermore, *G. vaginalis* biofilms tolerate higher amounts of hydrogen peroxide and lactic acid compared to planktonic cultures [15].

The high rate of BV recurrence may thus be explained by the presence of a biofilm protecting the bacteria from antibiotic treatment, moreover, serving as a reservoir for regrowth of pathogens [8]. Antibiotics (metronidazole or clindamycin) are the treatment of choice against BV and have high initial cure rates in clinical trials [16, 17]. Alternative strategies, including antiseptics, disinfectants, and vaccines, have been tested, but evidence regarding their effectiveness is mixed [11, 18]. Vaginal oral probiotics with or without prebiotics or concomitant antibiotics, and acidifying agents have also been explored as adjuvants after therapy [16, 19–21]. Thus, up to 50% decrease of recurrences seems to be possible, but novel approaches to prevent recurrence are urgently needed [22].

To attack biofilms of *G. vaginalis* and thus potentially prevent recurrence, we have screened a variety of compounds and found that amphoteric tensides such as cocoamphopropionate, administered alone or in combination with metronidazole, were very effective in disrupting *G. vaginalis* biofilms in vitro [23]. Here, we report a randomized double-blind controlled clinical trial which investigated the tolerability of cocoamphopropionate administered as a pessary after initial metronidazole treatment of BV. Moreover, we compared the effectiveness of cocoamphopropionate versus commercially available lactic acid pessaries against BV recurrence. The vaginal microbiota, clinical parameters, and biofilm characteristics were analyzed for a period of 3 months. A cohort of 20 women recruited independently of the clinical study served as control for the healthy vaginal flora. We compared the microbiota composition in woman with and without biofilms and identified biomarkers for the presence of biofilms in BV. The influence of metronidazole and pessaries on microbial communities was investigated. The time series of five consecutive visits allowed us to analyze in detail which clinical parameters and microbiota characteristics might affect recurrence.

## Methods

### Study design

We performed a prospective parallel-design, double-blind, randomized, controlled investigation. The vaginal pessary WO 3191 (certified medical device) was compared to lactic acid pessaries (certified medical device, Dr. August Wolff GmbH & Co. KG Arzneimittel, Germany) with respect to safety, tolerability, and efficacy following treatment of BV with metronidazole. Vaginal fluid and urine samples were obtained from all subjects in the clinical trial and from a cohort of healthy women that served as a control for the healthy vaginal microbiota. WO 3191 pessaries contained the amphoteric tenside cocoamphopropionate and, like lactic acid pessaries (LAP), contained lactic acid and sodium lactate. As both products looked similar, they could be assigned randomly in a blinded manner. The study protocol was approved by the local ethics committee (Bayerische Landesärtzekammer, München), and written consent was obtained from all participants. This study was conducted in accordance with the Declaration of Helsinki on Ethical Principles for Medical Research Involving Human Subjects. Principles and guidelines for good clinical practice were followed. The study was registered on ClinicalTrials.gov with the identifier NCT02687789 and in the Eudramed database with the identifier CIV-13-12-011731. The study protocol was approved by the local ethics committee of the Medical Association of North Rhine, Duesseldorf, Germany (Application number 00008571).

The study design and number of patients analyzed in each visit is shown in Additional file 1: Figure S1 as a Cochrane diagram. Study participants received 2 g of metronidazole (single dose) orally (visit 1). At visit 2 after 7 to 28 days, they were randomly assigned to receive either WO 3191 or LAP. Each pessary was applied intravaginally by the study participants for 3 weeks, twice per week. An intermediate examination took place after 1 week (visit 3) and the final examination after another 2 weeks (visit 4). A follow-up examination (visit 5) was scheduled 12–14 weeks after visit 4. Of the 44 included women, 34 completed the trial. The first database lock was performed after the last patient had completed visit 4, meaning that data entries and data management had to be performed before the database lock and unblinding of the data. One woman received metronidazole at visit 2 and was therefore excluded from the analysis. At each visit, vaginal fluid and urine were collected for the analysis of extracellular polymeric substances (EPS), biofilms, and microbiota composition and clinical symptoms were determined. Vaginal fluid of 20 healthy women was sampled independent of this clinical study as part of their gynecological routine examination and served as control group. These women provided informed consent and were not tested for biofilms, EPS in urine, or Nugent score.

### Assessment of biofilm in urine and vaginal samples

The method for EPS detection in urine [24, 25] was modified for quantitative measurements of EPS with an enzyme-linked lectin assay using peroxidase-coupled lectins. Briefly, urine was diluted 1:20 to 1:40 in PBS and 100-μl diluted urine was incubated in a 96-well polystyrene microtiter plate over night at 4 °C for attachment of dissolved EPS. Urine was discarded and the plates washed twice with 200 μl PBS. Horseradish peroxidase (HRP)-coupled lectin (wheat germ agglutinin (WGA)) bound to the EPS for 90 min. Residual HRP-WGA was removed by washing eight times with 200 μl PBS. As HRP substrate, 100 μl colorless 3,3′,5,5′-tetramethylbenzidine (50% (*v*/*v*) in PBS) was used, which was converted into a blue dye. This reaction was stopped after 10 min by addition of 0.5 M H_2_SO_4_ which resulted in a change of color to yellow. Optical density (OD) was measured at 450 nm and EPS considered positive when OD_450nm_ > 0.25.

For detection of biofilms, vaginal fluid was stained with 0.2% crystal violet in 100% ethanol and inspected under the light microscope (Olympus BX60, Germany). If more than 10 out of 50 vaginal epithelial cells (20%) were covered by a layer of bacteria, these patients were regarded biofilm positive. This cut-off was chosen based on the Amsel criteria [10].

### Study endpoints

The primary endpoint was local tolerability of medical devices defined as a cumulative sum score of solicited local (vaginal) adverse device events (ADEs, subjective symptoms and objective findings) between visit 2 and visit 4 (Table 1). Secondary tolerability endpoints included solicited and unsolicited adverse events and ADEs, global assessment of tolerability and leucocytes in vaginal smear. Secondary efficacy endpoints included assessment of the combined parameter of vaginal biofilm + EPS in urine (biofilm/EPS), percentage of patients with clue cells, changes in vaginal pH values, changes in Nugent score, changes in vaginal flora, global judgment of patient and investigator and recurrence of BV during the 12-week follow-up phase.

**Table 1:** Summary of study endpoints

A		
Primary endpoint	Secondary endpoints	
Local tolerability of medical devices (defined as a cumulative sum score of solicited local ADEs between visit 2 and visit 4)	Secondary tolerability endpoints: solicited and unsolicited adverse events and ADEs global assessment of tolerability leucocytes in vaginal smear	
	Secondary efficacy endpoints: assessment of biofilm/EPS percentage of patients with clue cells changes in vaginal pH values changes in Nugent score changes in vaginal flora global judgment of patient and investigator recurrence of BV during the 12-week follow-up phase	
B		
Excluded women (*n* = 72)	Included women (*n* = 44)	
No biofilm and/ or No EPS and/ or Nugent score < 7 and/ or other reason (e.g., smoking)	Age (mean)	32.4 years (range 19–51 years)
	Ethnicity	83.7% Caucasian, 11.6% with African descent
	Previous vulvovaginal diseases	39.5% (23.3% BV, 2.3% candidiasis, 16.3% unidentified vaginal infection or dybiosis)
	Miscarriage or preterm birth	7.0%
	Systemic hormonal contraceptives	48.8%

*ADE* adverse device event

### Sample collection and transport

Vaginal fluid was obtained by infusing 2 ml of saline solution into the vagina followed by rotation against the vaginal wall with a speculum and then collecting the vaginal fluid with a syringe. One third of the vaginal fluid (approximately 700 μl) was immediately transferred to a tube containing 2 ml RNAprotect (Qiagen, Germany), one third was transferred to an empty tube for microscopic analyses of the biofilm and one third was transferred to an empty tube for Nugent score assessment. All tubes were immediately frozen at −20 °C. They were transported at −20 °C within a week and finally stored at −70 °C. Midstream urine for EPS analyses was collected by the study subjects in standard tubes and then stored and shipped at 4 °C within 3 days.

### DNA extraction

DNA was extracted from vaginal fluid suspension using the peqGOLD Tissue DNA Kit (Peqlab, Germany) with pretreatment and modification: Vaginal fluid (250 μl) was centrifuged at 13,000 rpm for 5 min. Pellets were resuspended in 700 μl lysis buffer, 15 μl RNase, and 20 μl Proteinase K from the Tissue DNA Kit. Cell lysis was obtained by adding this suspension to 0.5 g of silica beads which were covered with 500-μl ice-cooled phenol. The bead-suspension mix was shaken at 5 m/s for 1 min in three intervals which were 2 min apart using the MO-BIO PowerLyzer™ (Mo Bio Laboratories, USA). After centrifugation for 1 min at 13,000 rpm, the upper phase containing the DNA was further processed according to the manufacturer’s instructions of the peqGOLD Tissue DNA Kit starting with DNA binding.

### Preparation of 16S rRNA gene amplicon libraries, sequencing, and data processing

Amplicon library preparation for high-throughput sequencing on a MiSeq Illumina platform (280 bp paired end chemistry) was performed as previously described [26]. Barcoded amplicons of the 16S rRNA gene V1-V2 regions were sequenced and a total of 12,495,036 raw reads obtained. Only the read pairs with overlap longer than 30 bp and sequence length longer than 300 bp after merging were taken into account. Then, the primers in sequences were trimmed using cutadapt [27]. Subsequently, sequences with the number of expected errors above 1 were removed using VSEARCH [28]. After quality filtering, primer trimming, and merging pairs, 6,757,844 reads were obtained.

As an example, the difference between the 97 and 99% similarity threshold was analyzed for *Sneathia sp*. and the full table is provided in Additional file 2: Table S1. We could show that the read number does not change for the most abundant operational taxonomic units (OTUs) which we focused our analysis on (Additional file 3: Figure S2B). Therefore, clustering was performed based on a 97% similarity threshold. Chimeras were removed by using the RDP database (RDP_trainset15_092015.fa) [29]. All steps were performed with VSEARCH [28]. Only those OTUs that were present in an abundance > 0.001% of the whole experiment were considered, resulting in 319 OTUs and a total of 6,438,017 reads. All OTUs were analyzed using stand-alone blastn against the vaginal 16S rDNA reference database (STIRRUPS) [30]. For taxonomic assignment at the species level, 97% identity or higher was used. For OTUs that could not be assigned to a species, Bayesian classification using the Ribosomal Database Project Classifier with a confidence threshold of 80% was applied resulting in a taxonomic classification to the genus level or higher taxa [31]. Seven OTUs were identified as contaminants and removed from the dataset since they were highly abundant only in samples from healthy women which were collected separately from the randomized controlled trial in a different clinic, are not members of the vaginal microbiota, and have been identified as contaminants before (Additional file 2: Table S1, Additional file 3: Figure S2A) [32]. These OTUs are usually found in soil or water, and one contaminant (*Streptococcus mutans*) was acquired in the laboratory where work focuses on research on this species [33–39] and where also DNA was extracted for this study. For the phylogenetic tree, OTUs were aligned to sequences from the vaginal 16S rDNA reference database using MUSCLE. The tree was constructed with the neighbor joining method using MEGA6 [40]. Additionally, clustering was performed on a 99% similarity threshold, and all following steps were performed as described above.

### Statistical analysis of 16S rRNA gene sequences

Microbiota analyses were performed for all screened women and healthy controls. OTU data were rarefied to 979 reads, and all subsequent analyses were performed on this dataset. Resampling efficiency was determined based on the standard error (standard deviation/mean) after resampling 20 times. Rarefaction curves were obtained with the vegan package in the R environment [41, 42]. Shannon index (H′) was determined on the OTU level, and boxplots and cumulative dominance plots showing the ranked species abundance were created in PRIMER7 with the PERMANOVA+ add-on software (159 PRIMER-E, 1). Mean values and standard deviations were calculated and the Wilcoxon rank sum test used for statistical analyses.

For the principal coordinate analysis (PCO), a resemblance matrix was generated using the Bray-Curtis coefficient in PRIMER-E7. For the linear discriminant analysis (LDA) effect size (LEfSe) pipeline, alpha values of 0.05 for the factorial Kruskal-Wallis test and a logarithmic LDA score threshold of 2.0 were used [43].

### Statistical analysis of the clinical data

Explorative statistical tests were performed using a two-sided alpha level of 5 and 10%, and the respective 90 and 95% CIs were calculated. The number of observations (N), mean standard deviation (SD), minimum, 1st quartile (Q1), median, 3rd quartile, and maximum were calculated separately for each arm for continuous (or semi-continuous) data. Additionally, categorical data were displayed separately for each treatment group using absolute frequencies and percentages (%) and time-to-event data were described by medians and quartiles calculated by Kaplan-Meier life-table methods [44]. Correlations between diagnostic parameters, such as biofilm, EPS, and clue cells were calculated using the phi coefficient, and significance was determined using Fisher`s exact test.

### Light and field emission scanning electron microscopy

For field emission scanning electron microscopy, samples were fixed with 2% glutaraldehyde and 5% formaldehyde in HEPES buffer (HEPES 0.1 M, 0.09 M sucrose, 10 mM CaCl_2_, 10 mM MgCl_2_, pH 6.9) and stored at 7 °C overnight. Cover slips with a diameter of 12 mm were coated by placing 50 μl of poly-l-lysine solution (Sigma, Germany) for 5 min on the cover slips, washed in distilled water and air-dried. Fifty microliters of the fixed samples were placed on a cover slip and allowed to settle for 10 min. Cover slips were then fixed in 1% glutaraldehyde in TE-buffer (20 mM TRIS, 2 mM EDTA, pH 7.0) for 10 min at room temperature and subsequently washed with TE-buffer before dehydrating in increasing concentrations of acetone (10, 30, 50, 70, 90, 100%) on ice for 10 min for each step. Samples were then subjected to critical-point drying with liquid CO_2_ (CPD 300 Leica, Germany). Dried samples were covered with a gold-palladium film by sputter coating (SCD 500 Bal-Tec, Liechtenstein) before examination in the Zeiss field emission scanning electron microscope Merlin using the Everhart Thornley SE detector and the inlens SE detector in a 50:50 ratio with an acceleration voltage of 5 kV. Light microscopic images were obtained with an Olympus BX60 microscope after staining of samples with 0.2% CV. Images were recorded with the Olympus DP73 digital camera.

## Results

### Clinical study

A prospective, double-blind, randomized clinical study was performed to analyze the safety, tolerability, and effectiveness of two pessaries on clinical parameters, biofilm presence, microbiota composition, and recurrence after metronidazole therapy of BV.

### Study participants

One hundred sixteen premenopausal women, who suffered from discharge and fishy smell, were investigated by their gynecologists and included in the study if they were BV positive by three out of four Amsel criteria (gray-white discharge, malodor after application of 10% KOH - solution, pH > 4.5 or the presence of at least 20% clue cells). Additionally, they had to fulfill the following inclusion criteria: Nugent score > 6 (blinded performed by an independent investigator), positive for extracellular polymeric substances (EPS) in urine, and a biofilm on vaginal epithelial cells. These analyses were performed to allow the differentiation between biofilm with EPS and those with dissolved EPS.

Of 116 premenopausal patients with BV (diagnosed by Amsel criteria), 44 were randomized and included in the clinical study based on the established inclusion criteria. Seventy-two women were excluded due to EPS score, biofilm absence, low Nugent score, or other reasons despite clinical diagnosis of BV by the investigating gynecologists (Additional file 4: Figure S3A). The difference in EPS score, biofilm, and Nugent score between included and excluded patients was highly significant (*p* = 5.4 × 10^−7^, *p* = 0.0005, and *p* = 0.0007; Additional file 4: Figure S3A).

The mean age of the included patients was 32.4 years (SD ± 9.6) and ranged between 19 to 51 years. Most patients were Caucasian (83.7%) and 11.6% of patients were of African descent. With respect to previous vulvovaginal diseases, 39.5% had already experienced some kind of infection or infestation (23.3% BV, 2.3% candidiasis , and 16.3% unidentified vaginal infection or dysbiosis). Seven percent had experienced a miscarriage or preterm birth previously. The most commonly used concomitant medications were systemic hormonal contraceptives (48.8% of patients). At the second visit, 26 women were randomly assigned to receiving LAP and 18 women to receiving WO 3191. Of the 44 patients who were randomized to either of the two pessary treatments, 43 started and 38 finished treatment (patients reached visit 4) and 34 stayed in the study until follow-up.

### The microscopy of clue cells, biofilm, and EPS

**Fig. 1:**
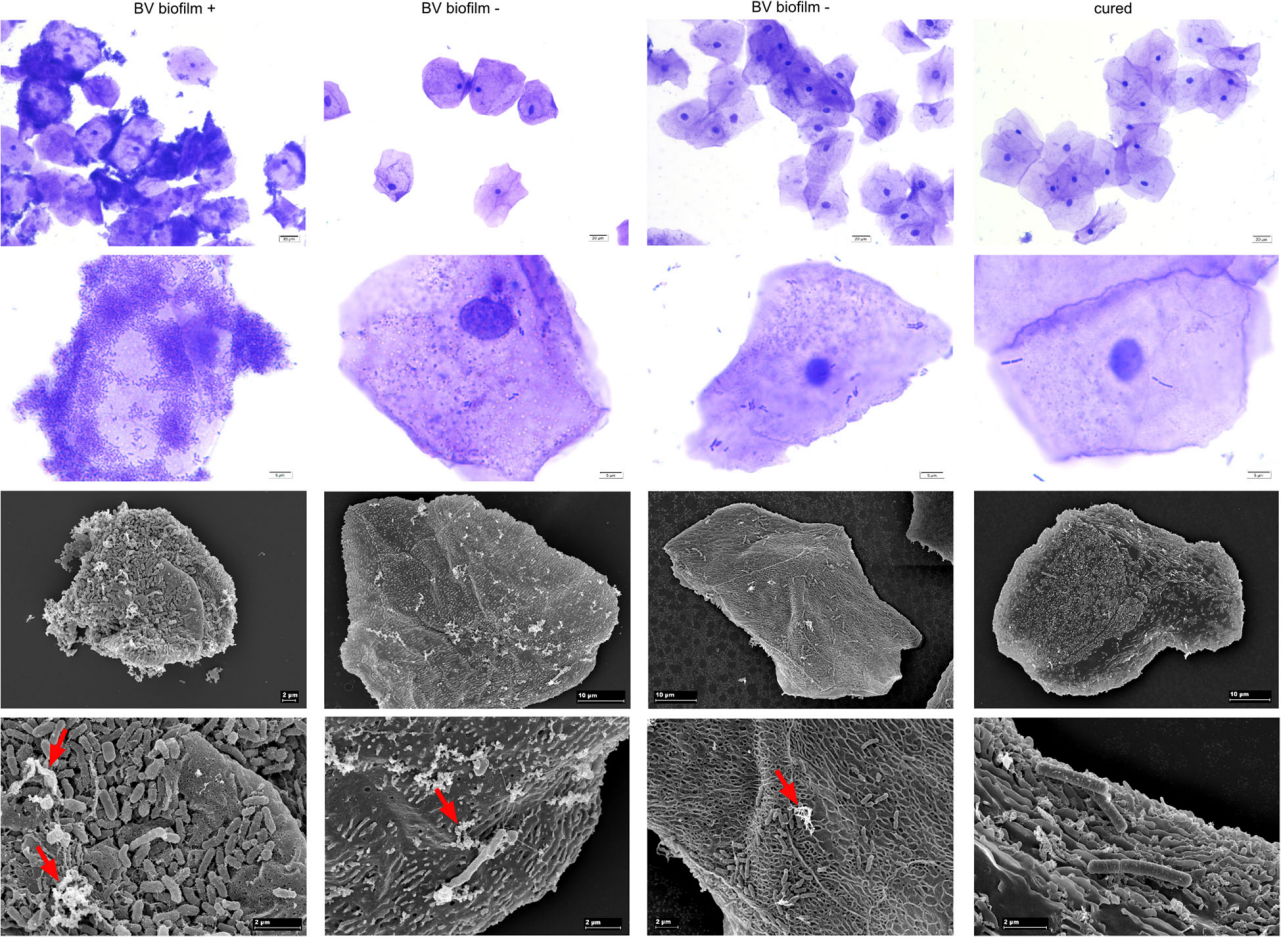
Microscopic images of vaginal epithelial cells with attached biofilms. From left to right: Vaginal epithelial cells from patients with BV either with (BV biofilm +) or without biofilm (BV biofilm −) and after metronidazole treatment (cured). Top two rows: light microscopy of samples stained with crystal violet. Bottom two rows: scanning electron micrographs. Red arrows indicate EPS.

Biofilm was microscopically determined on epithelial cells after CV staining to visualize them more clearly. Light and scanning electron microscopy showed clue cells which are biofilm covered vaginal epithelial cells during BV that contained different morphotypes of bacteria. EPS was clearly visible and biofilm covered most of the epithelial cell surface (Fig. 1). Epithelial cells of some women with BV were not covered by biofilms and had only very few attached bacteria which also were of different morphotypes (likely not *Lactobacillus* sp*.*). However, these epithelial cells also had residues of EPS on the surface. In the samples of cured women, *Lactobacillus*-like morphotypes were present and no biofilm was formed. Correlations between biofilm, EPS, and clue cells which were determined by the gynecologist, were identified in the complete patient data set. A positive correlation between clue cells and the bacterial biofilm could be observed at visit 2 (phi = 0.32; Fisher’s exact test: *p* = 0.1171), demonstrating that if clue cells were present, it was likely that a bacterial biofilm could also be detected. The correlation increased and reached significance at visits 3 and 4, and it persisted until visit 5 (visit 3: phi = 0.42; *p* = 0.057; visit 4: phi = 0.48, *p* = 0.031; visit 5: phi = 0.49, *p* = 0.016).

Additionally, a weak positive correlation between clue cells and the combined parameter of bacterial biofilm + EPS in urine (biofilm/EPS) was observed at visit 4 (phi = 0.46; *p* = 0.013).

### Tolerability of pessaries

WO 3191 did not lead to irritation and was well tolerated, with 88.9% of patients judging the global tolerability to be good/very good on a 6-point rating scale (Additional file 4: Figure S3B). Investigators determined the global tolerability of WO 3191 to be good/very good in 83.4% of patients. The mean scores of global tolerability for WO 3191 and LAP were not significantly different when assessed by patients (*p* = 0.9539) or investigators (*p* = 0.4532). Local tolerability was assessed by the intensity of solicited adverse device events (Additional file 4: Figure S3C). The majority of BV patients was free from vaginal symptoms after treatment with metronidazole (visit 2, baseline for treatment with pessaries). During the 3-week double-blind treatment period (visits 3 and 4), no significant differences concerning local tolerability between treatment groups were observed. The mean sum scores for both subjective symptoms and objective findings were low in both treatment groups. No safety concerns were identified with WO 3191 or LAP. The most commonly reported adverse event was bacterial vaginosis with a comparable occurrence in both treatment groups.

### Changes in biofilm EPS status, vaginal pH, and Nugent score

While initially all patients were biofilm/EPS positive due to the established inclusion criteria (*N* = 44), this fraction decreased continuously during the study. In the WO 3191 treatment group (*N* = 15), 40.0% of patients were biofilm/EPS positive at visit 2 (baseline), 26.7% at visit 3 and 6.7% at visit 4. In the LAP treatment group (*N* = 22), the percentage of patients being biofilm/EPS positive initially decreased from 27.3% at visit 2 to 18.2% at visit 3 and thereafter increased to 36.4% at visit 4.

Vaginal pH levels increased slightly in both treatment groups during the double-blind treatment phase. With respect to the Nugent score, more WO 3191 recipients were classified as “healthy” (Nugent score 0–3) at visit 4, compared with LAP recipients (86.7% of *N* = 15 vs 77.3% of *N* = 22), but this difference was not significant. To summarize, although we gained some significant result in favor for WO 3191 (regarding net number of patients profiting regarding their biofilm EPS status) the small number of patients did not allow to conclude that one pessary is clinically more effective than the other in the post treatment of BV (Additional file 5: Table S2).

### Microbiota analysis

For microbiota analyses of vaginal fluid, 60 samples of women with acute BV from the excluded group were analyzed as well as those samples of women with acute BV who completed the clinical study until visits 4 or 5. In total, 96 samples were analyzed and compared to samples from 20 healthy women. The number of samples used for microbial analysis per visit and treatment group is depicted in Additional file 1: Figure S1. These numbers were smaller than the numbers used for analysis of clinical outcomes as microbial samples were not available.

### Taxonomic affiliation of reads

After abundance filtering and contamination removal, 312 OTUs and a total of 6,363,304 V1-V2 ribosomal DNA reads (mean per sample = 24,857 ± 23,782 reads) were obtained. A full OTU table is available in Additional file 2: Table S1. Forty-seven percent of the sequences could be assigned to the species level, 33% to the genus level, and 20% to the family level or higher taxa. To validate the species level taxonomic affiliation, the genus *Lactobacillus* was used as an example because of its importance in the vaginal microbiota.

### Rarefaction and sequencing depth

The full-length 16S rRNA gene sequence of all species within this genus that were available in the STIRRUPS 16S rRNA vaginal database was used for constructing a phylogenetic tree. All OTUs that were assigned to this genus were added to the tree. The phylogenetic tree showed that OTUs identified as species are phylogenetically closest to the species they were assigned to (Additional file 6: Figure S5). In our dataset, we could observe a strong difference regarding the sequencing depth, where samples ranged from 979 to 213,795 reads. A rarefaction analysis showed that the diversity was still high within the samples. Some had almost reached saturation while for others, a higher sequencing depth would have been needed for saturation (Additional file 7: Figure S6A). To be able to compare all communities with each other, OTU abundance was rarefied to 979 reads in all subsequent analyses. A PCO showed that samples with < 5000 reads did not differ from the ones with higher sequencing depth in terms of community composition (Additional file 7: Figure S6B). Although they were few, we did not exclude these samples because we did not want to further minimize the study size. Resampling led to underrepresentation of α-diversity and limits the analysis to comparisons within this dataset. To evaluate whether resampling once would be sufficient for this analysis, we resampled five random samples with low, average, and high sequencing depth 20 times and calculated the standard error (Additional file 6: Figure S6C). We could show that resampling affects only low abundant OTUs below 1% of relative abundance. Because the error was low for low abundant OTUs and our study focuses on high abundant OTUs, resampling this dataset once was sufficient.

### Microbial diversity of vaginal fluid samples

**Fig. 2:**
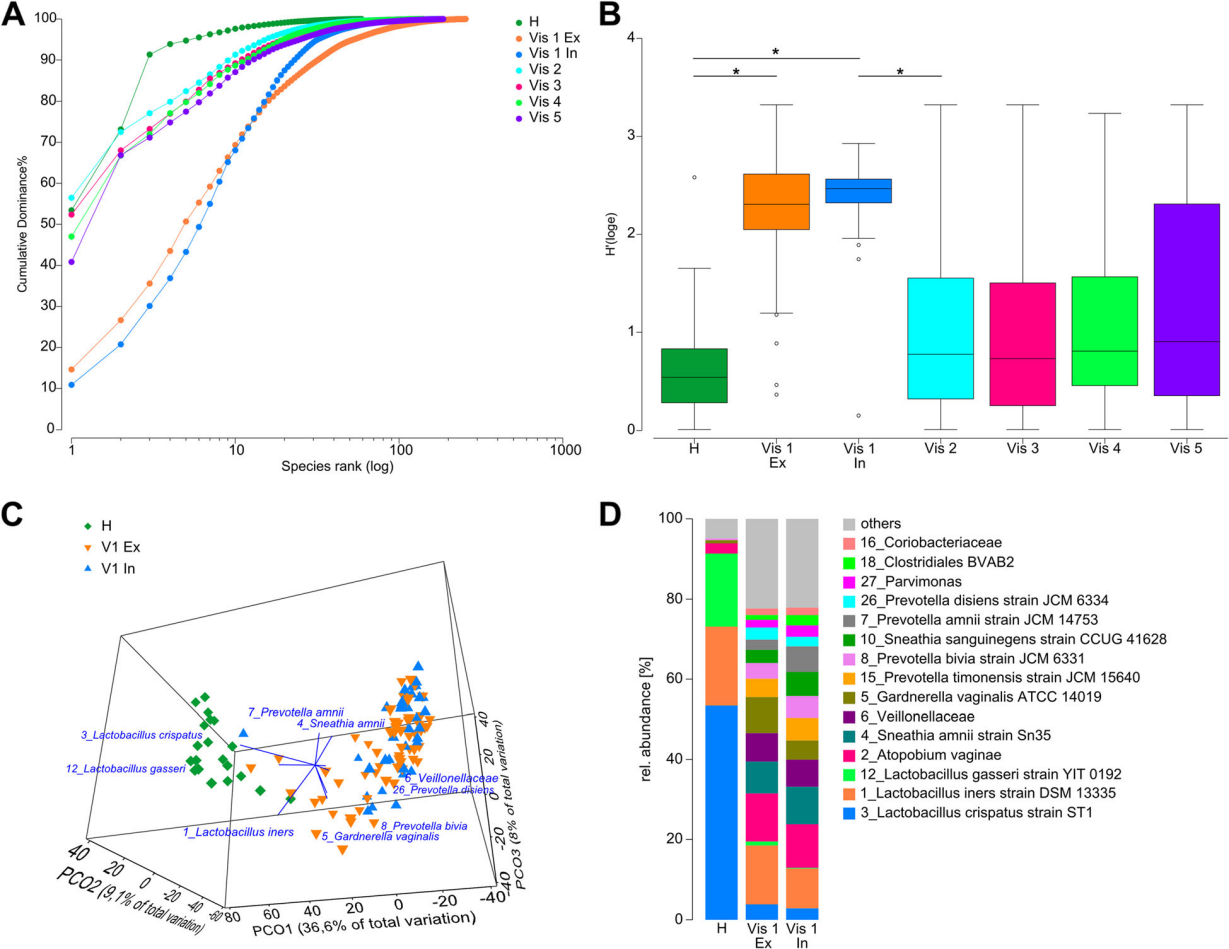
Rank abundance and Shannon diversity of vaginal microbiota. **a** Dominance plot of cumulated samples from all visits. H health, vis/v visit, ex excluded, in included. **b** Shannon indices of all groups. Mean and quartile range are shown. Asterisks indicate significant (*p* < 0.01) differences assessed by a Wilcoxon rank sum test. **c** PCO of healthy women and included or excluded women with acute BV. **d** Cumulated abundances for each group. OTUs below 1% relative abundance are summarized as others.

Microbial diversity was very low in the samples from healthy women. Three OTUs were sufficient for 90% of cumulative dominance, and a species rank of 59 was reached (Fig. 2a). In contrast, diversity of vaginal fluids of women with BV was very high. In the included group, 23 OTUs were needed for 90% cumulative dominance and a total of 255 OTUs identified. In the excluded BV group, 32 OTUs covered 90% cumulative dominance and a total of 175 OTUs were identified. After treatment with metronidazole, vaginal fluid from former BV patients showed a low diversity just like that of healthy women. Because not all women responded similarly to metronidazole and a few remained with a high diversity, 9 OTUs were needed for 90% cumulative dominance and an OTU rank of 140 was reached. Over time, at visits 3, 4, and 5, overall diversity increased again, until at visit 5, 13 OTUs covered 90% cumulative dominance and a total of 186 OTUs could be identified. Shannon diversity (Fig. 2b) increased significantly from health (H′ = 0.67 ± 0.59) to BV (H′_Ex_ = 2.24 ± 0.57, H′_In_ = 2.39 ± 0.46) as was assessed by a Wilcoxon rank sum test. After metronidazole treatment, it decreased again (H′_Vis 2_ = 0.94 ± 0.72). The interquartile range (IQR) was higher after metronidazole treatment than in healthy women due to large individual differences in response to treatment. H′ remained stable until visit 5, and the IQR increased even further.

### *L. crispatus* dominates healthy community state types (CSTs) whereas BV is characterized by high diversity and several CSTs

A principal coordinate analysis (PCO) showed that microbiota of vaginal fluid from healthy individuals were clearly different from those of BV patients. This was caused by *L. crispatus* in healthy women and a group of several OTUs in BV patients. All women with BV clustered together, regardless of their inclusion in the main study. However, some samples overlapped with healthy controls, with groupings driven by *G. vaginalis, Prevotella bivia*, and *L. iners* (Fig. 2c). Samples of women with BV that clustered closest to the healthy group were derived from women colonized by *L. crispatus*.

The overall vaginal microbiota composition of the healthy control group was dominated by three species of *Lactobacillus*, namely *L. crispatus*, *L. iners*, and *L. gasseri*, with a cumulative relative abundance of 61, 25, and 10% each (Fig. 2d). Accordingly, individual microbiota from healthy individuals clustered into three community state types that were each dominated by *L. crispatus*, *L. iners*, and *L. gasseri*, with a ratio of 2.5:1:1, respectively (Additional file 8: Figure S6). The most abundant species of excluded and included women with BV were *L. iners*, *A. vaginae*, and *Sneathia amnii.* Although these species were differently distributed among individual women of each group (Additional file 9: Figure S6A), their cumulative abundance was not significantly different (Wilcoxon rank sum test; Fig. 2d). Whereas *L. iners* was also abundant in healthy women, *A. vaginae* could rarely be found in them (rel. abundance = 0.8%). None of the healthy women were colonized with *S. amnii*. Differences between both groups could be observed for *G. vaginalis* which was less abundant and *S. sanguinegens* which was more abundant in included women (*p* < 0.01). Inter-individual differences were stronger in BV patient samples. Communities were dominated by either *L. iners*, *P. bivia*, *S. amnii*, or *P. amnii* in both groups. Vaginal fluids of two women were dominated by *Lactobacillus* sp., even in BV (Additional file 9: Figure S6).

Biomarkers for health and BV were identified using LEfSe (Additional file 8: Figure S7). Included and excluded patients were analyzed separately. The best markers for health were in both cases *L. crispatus* and *L. gasseri.* The best markers for BV were also similar in both groups. The highest LDA score was determined for *A. vaginae* and *S. amnii.* They were followed by *G. vaginalis*, an OTU of Veillonellaceae, *P. timonensis, P.amnii* and other *Prevotella* species and *S. sanguinegens,* which were located at different ranks in the excluded and included group but present in both.

### *S. sanguinegens* is a biomarker for biofilms in BV

**Fig. 3:**
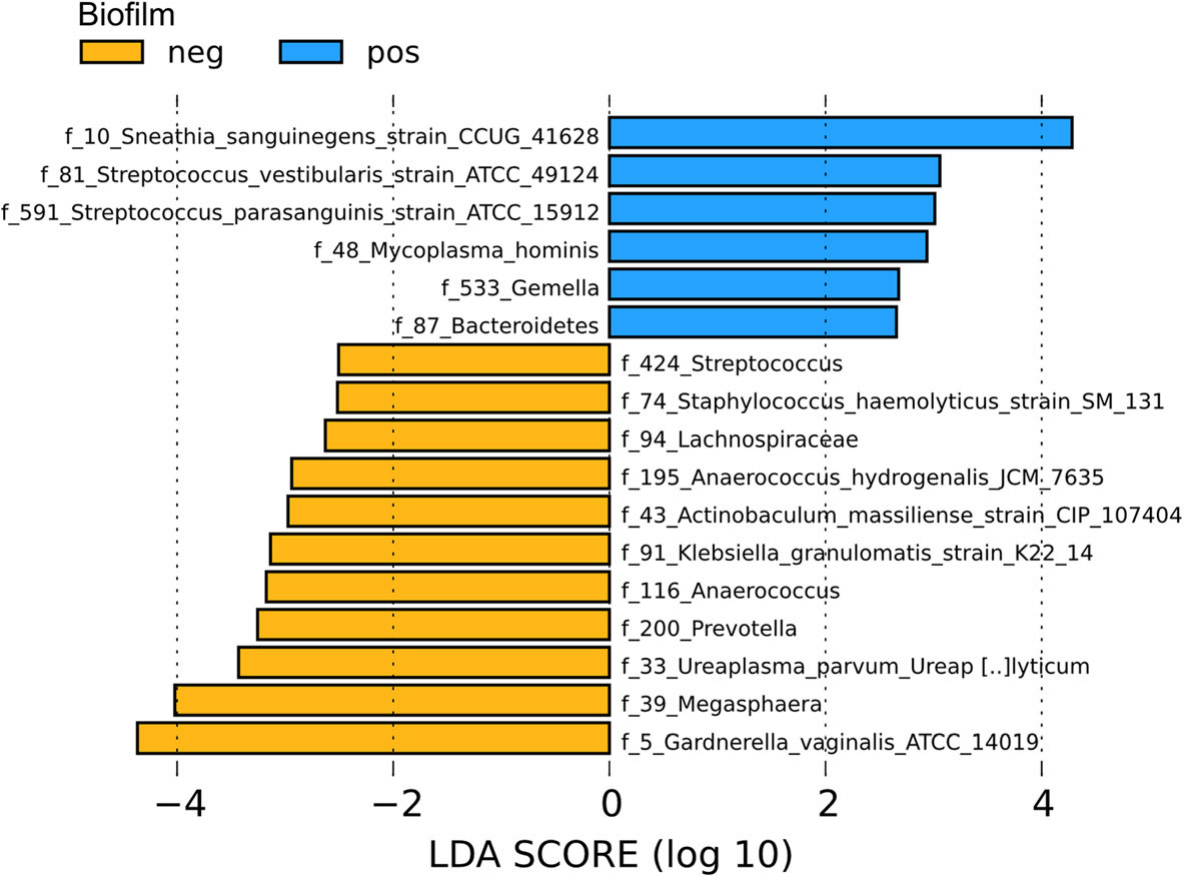
Biomarkers for biofilms in BV. LEfSe biomarker analysis comparing all samples from BV (visit 1) according to biofilm status (LDA threshold = 2.0).

We found that 14% (*n* = 15) of all women with BV did not have a biofilm. We therefore compared their microbiota to those of the patients with biofilms (86%, *n* = 91) (Fig. 3). LEfSe analysis revealed *S. sanguinegens* as the top biomarker for biofilm presence. Surprisingly, *G. vaginalis* and *Megasphaera* sp., both commonly identified in BV, were identified as the top two biomarkers for biofilm absence.

### Metronidazole causes a shift in community state type

**Fig. 4:**
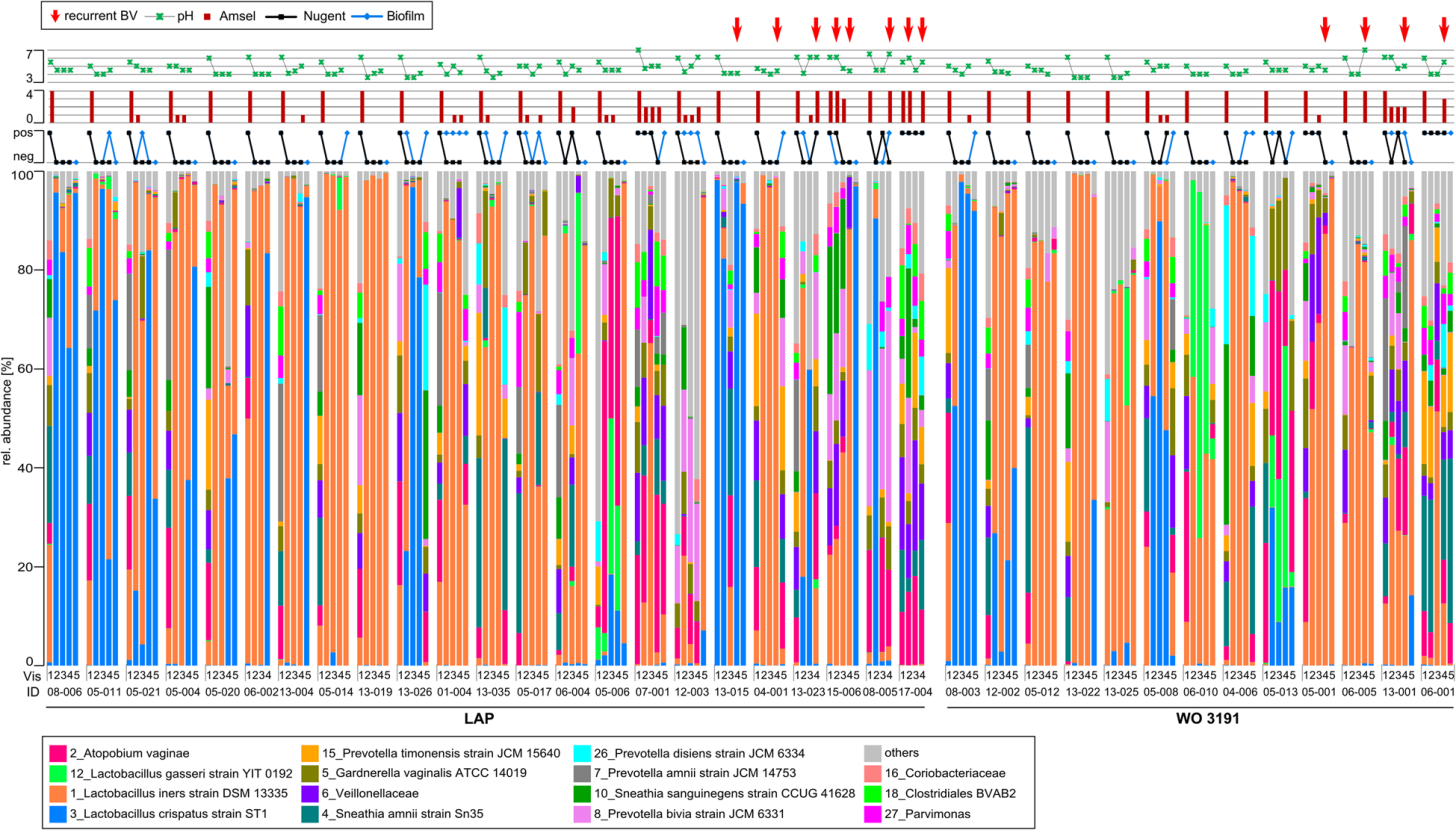
Composition of the vaginal microbiota of all women included in the study. Patients are separated according to treatment (LAP vs. WO 3191). Visits (1–5) and corresponding data (Nugent score, biofilm status, number of Amsel criteria and pH) are shown *above* and *below*. *Red arrows* indicate BV recurrence. OTUs below 1% abundance are summarized as “others”. LAP = lactic acid pessaries.

**Fig. 5:**
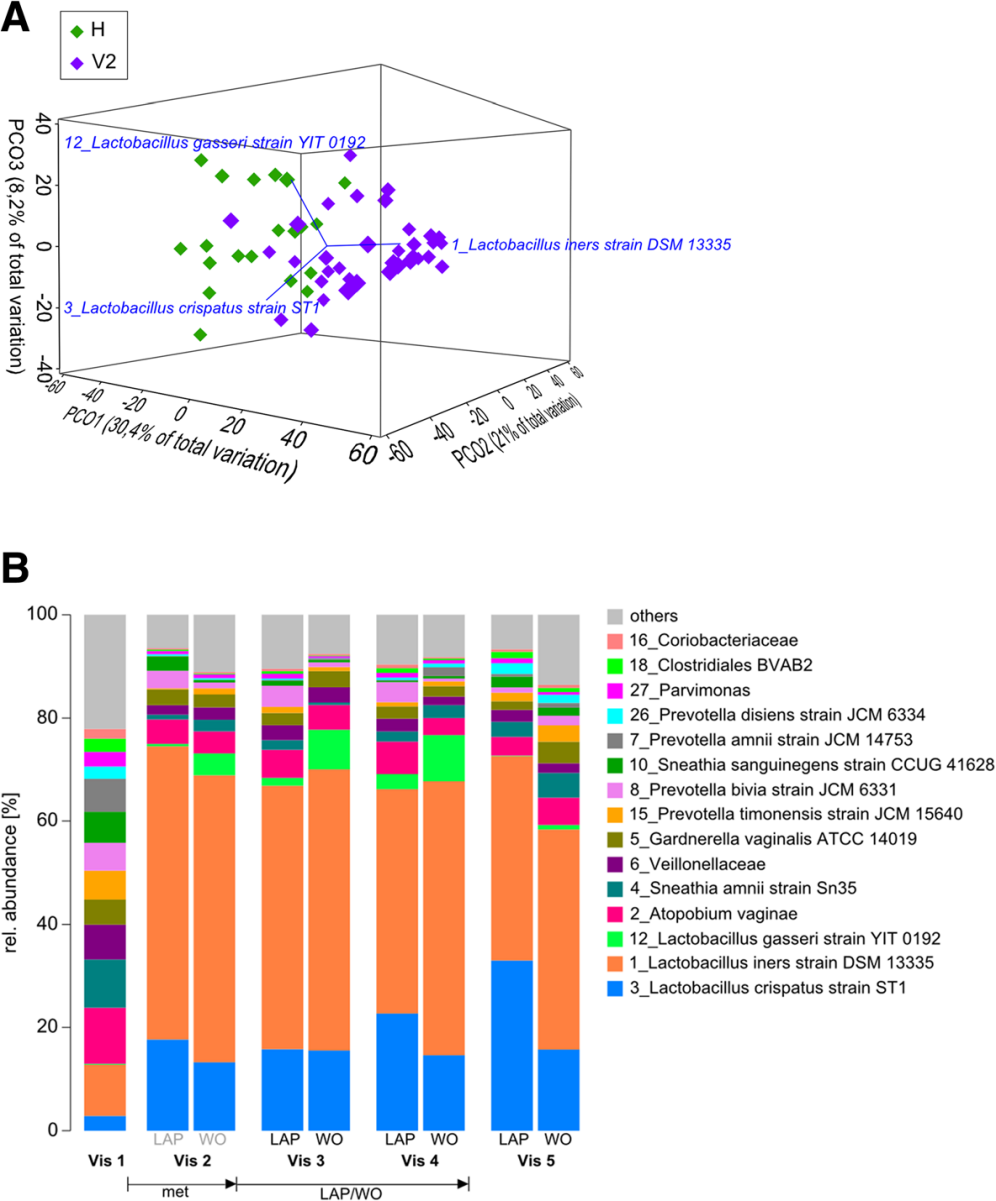
The effect of metronidazole and pessaries. **a** Principal component analysis of the vaginal microbiota composition in healthy women and women after metronidazole treatment. Vectors indicate the species driving variation. H healthy cohort, V2 women from the clinical study at visit 2 (after metronidazole treatment). **b** The microbiota response to pessaries. Cumulated microbiota composition for LAP and WO 3191 treatment for all visits. At visit 1, metronidazole was provided. At visit 2, women were randomly assigned to one of the two pessary treatments. From visits 3 to 5, they had been treated with the respective pessary. OTUs *below* 1% abundance are summarized as “others”. LAP = lactic acid pessaries

Inter-individual variability after treatment with metronidazole was high (Fig. 4). The microbiota of the women was affected in different ways by the antibiotic. In 23 of 36 patients of the microbiota analysis (M-BA) population (64%), it resulted in low diversity and colonization with *Lactobacillus* sp*.* concurrent with lack of symptoms (Amsel criteria), low pH, low Nugent score (NS), and the absence of biofilm. Eight women (22%) changed to a low NS but remained biofilm positive. In five of these, the vaginal microbiota was dominated by *Lactobacillus* sp., while it remained highly diverse in the other three. According to NS, five women (14%) did not respond to metronidazole treatment and those five women remained with H′ = 2.1 ± 0.36 and therefore a highly diverse microbiota (patients 07-001, 15-006, 17-004, 05-001, and 06-001; Fig. 4). Only one of these five women was biofilm negative (15-006) after metronidazole treatment and four remained biofilm positive. A PCO showed that the microbiota of women that did not respond to metronidazole treatment could not be distinguished from the responsive group at visit 1 (Additional file 10: Figure S8A). Identification of a biomarker at visit 1 which would have allowed the prediction of metronidazole success or failure was not possible (Additional file 10: Figure S8A). Interestingly, in 68% (*n* = 21) of the cured women, the *L. iners* CST was found (> 50% of cumulative abundance), whereas in only 17% the *L. crispatus* CST was found (Fig. 4). A PCO showed that the communities of healthy women clustered distinct from those of patients responsive to therapy and that these different clusters were driven by *L. crispatus* and *L. gasseri* for healthy women and *L. iners* for patients responsive to therapy (Fig. 5a, b) confirming the observed CST switch after metronidazole treatment.

Recurrence emerged in 28% (*N* = 10 of *N* = 36 in total) of study participants included in the microbiota analyses at either visit 4 or visit 5 (Fig. 4, red arrows). It occurred in four out of the five patients that did not respond to metronidazole therapy (based on NS), but also occurred in those six women that had initially responded. Of those, two (out of three) biofilm positive women with a diverse microbiota showed recurrence and four women with low diversity microbial communities and lack of clinical symptoms also showed recurrence. The presence of a biofilm was no indicator for recurrence. Women with or without biofilm showed recurrence of BV. PCO before recurrence could not separate recurrence/no recurrence, and LEfSe biomarker analyses did not produce conclusive results (Additional file 10: Figure S8B,C). However, women who developed BV at visit 4 or later (*N* = 10) had a higher diversity at visit 3 (H = 0.69 ± 0.69) than those who remained healthy (*N* = 26, H = 1.62 ± 0.77), and this difference was significant (*p* = 0.004). Recurrence rates for the clinical population were slightly lower due to a different population set. Between visits 2 and 4, three and five women experienced recurrence in the WO 3191 (*N* = 15) and the LAP group (*N* = 22), respectively. During follow-up, two of 11 in the WO 3191 and four of 19 in the LAP group had a recurrence of BV. However, this difference of recurrence was not statistically significant.

Most of the 26 women mentioned above without BV recurrence showed a stable microbiota composition dominated by *Lactobacillus* sp., low pH, and NS, and remained biofilm negative throughout the follow-up visits. *L. iners* remained the most abundant species, although six women shifted from *L. iners* towards *L. crispatus* as main colonizer over time. Ten women became positive for biofilm at some point throughout the study without further consequences, and six women returned to a highly diverse symptomless community at visit 5. All of these observations were independent of the pessary treatment groups.

### Post-antibiotic treatments did not influence microbial community composition

Upon treatment with LAP or WO 3191, only minor and nonsignificant microbiota shifts could be observed. At visit 2 (after antibiotic therapy and before application of pessaries), the cumulative abundance of *Lactobacillus* sp*.* (*L. crispatus*, *L. iners*, *L. gasseri*) was similar in both treatment groups (75% for Lactobacillus sp. (Vag) and 73% for Lactobacillus sp. (WO), Fig. 5b). After pessary treatment at visit 4, cumulative abundance of *Lactobacillus* sp*.* was higher in the WO 3191 treated group (77 vs. 69% in the LAP group, n.s.) which was due to a higher cumulative abundance of *L. gasseri* (9%, n.s.). At follow-up (visit 5), the cumulative abundance of *Lactobacillus* sp*.* had stayed stable in the LAP-treated group (73%) with a steady increase of *L. crispatus* (from 18% at visit 2 to 33% at visit 5 n.s.) whereas it had decreased in the WO 3191 group to 59% cumulative abundance (n.s.). The diversity was not significantly different between treatment groups at any time point. Individual microbial profiles of all women from visit 2 to visit 5 are shown in Additional file 9: Figure S6B.

## Discussion

The aim of our study was to gain clinical experience and further knowledge about the certified medical device vaginal suppository WO 3191 with respect to safety, tolerability, and efficacy in the post-treatment of bacterial vaginosis. We further determined whether WO 3191 would be superior to LAP for reducing biofilm-mediated recurrence of BV after metronidazole treatment. Eighty-six percent of all screened women with BV were biofilm positive in our study as was previously found in another study [9]. Treatment with metronidazole resulted in reduction of this biofilm in 67% of women of which most stayed biofilm undetected until follow-up at visit 5. This is in contrast to a study by Swidsinski et al. (2008) which demonstrated that biofilms were indeed first apparently eliminated, but restored frequently after treatment with metronidazole [9]. This discrepancy may have been caused by the threshold for the presence of biofilms which was used here. We considered only those samples biofilm positive in which a minimum of 20% of epithelial cells were covered by a layer of bacteria whereas Swidsinski et al. did not set a cutoff for their observations.

Recent in vitro data from our group showed that amphoteric tensides are able to disrupt biofilms and their surrounding EPS. Although in the present clinical study only a small number of patients was available we found that there was a significant difference between the two treatment groups regarding the impact on “biofilm EPS” Additional file 5: Table S2). However, clinical parameters did not reflect the difference of this parameter. However, none of the treatments prevented or noticeably postponed recurrence of BV. This is most likely due to BV being such a multifactorial disease [4]. To prevent recurrence, therefore, a combination of several means should be used. For example, probiotics may be effective [11, 21]. It could be worthwhile to explore the combination of WO 3191 or LAP with *Lactobacillus* probiotics as strategy against BV recurrence. However, sexually transmitted BV through unprotected intercourse should also be considered in finding strategies for the prevention of recurrent BV [45].

In our study, we have compared the microbiota of healthy women to the microbiota of women with acute BV and found *G. vaginalis* as biomarker for BV. Interestingly, when the BV group was divided into those women with (14%) and without a biofilm (86%), *G. vaginalis* was identified as a biomarker for biofilm absence. Therefore, women with BV were colonized by *G. vaginalis*; however, women with biofilm presence had significantly less *G. vaginalis*. This is in contrast to studies who showed that *G. vaginalis* is an abundant component of biofilms in BV [9, 46]. However, these studies specifically targeted *G. vaginalis* with FISH probes, and although they have found other species, too, they did not detect *Sneathia* sp*.* and most of the relevant *Prevotella* sp*.* which, as our study suggests, may be even more common than *G. vaginalis* in BV-related biofilms. Furthermore, *G. vaginalis* has a strong strain variation and not all *G. vaginalis* isolates form biofilms to the same degree [47]. In addition, the structure of *G. vaginalis* populations is diverse and presumably can consist of biofilm-formers, non-formers, or a mixture of the two [48]. Nevertheless, a limitation to our study is the selection of the 16S rRNA gene V1-V2 region for sequencing which has been shown to underrepresent *G. vaginalis* [49, 50].

*S. amnii* was identified as most important biomarker for acute BV, and *S. sanguinegens* could be identified as biomarker for biofilm presence in acute BV. Little is currently known about the genus *Sneathia*. It was described as a new genus when *Leptotrichia sanguinegens* was renamed to *S. sanguinegens* after having been isolated from human blood and amniotic fluid in 2001 [51]. In 2012, *S. amnii* was discovered in the vaginal tract as part of the Vaginal Human Microbiome Project [52]. Its biochemical characteristics were assessed and several potential virulence factors that may be relevant in BV, such as the adherence to epithelial cells, were described. Later, several studies associated *Sneathia* sp. with BV using molecular techniques [5, 53–56]. *S. sanguinegens* was frequently identified in amniotic fluid and is associated with early-onset neonatal sepsis and preterm labor [57–59]. Like *A. vaginae* and other BV-related species, *S. sanguinegens* is also associated with pelvic inflammatory disease and infertility [58, 60]. Overall, our study suggests a high relevance of the genus *Sneathia* in BV.

Our microbiota analysis showed that the vaginal fluid microbiota of healthy women is characterized by a very low diversity and three different CSTs which are dominated by *Lactobacillus* sp.. The most common *L. crispatus* CST was followed by the *L. iners* and *L. gasseri* CSTs, in accordance with previous reports [3]. By contrast, BV microbiota were characterized by high diversity with *L. iners*, *Prevotella bivia*, *S. amnii*, and *P. amnii* as the most abundant species. In accordance with these results, Dols et al. suggested to diagnose BV by a combination of the absence of *Lactobacillus* sp*.* and a high diversity [57]. However, solutions for a molecular-based BV diagnosis are so far ambiguous. Two studies found that inclusion of Lactobacillus testing did not improve accuracy for BV diagnosis [54, 61], and other studies found only BV associated species to be indicative for the emergence of BV [54, 62].

After treatment with the antibiotic, patients exhibited low diversity *Lactobacillus* dominated vaginal microbiota; however, the *L. iners* CST was found in most women rather than the *L. crispatus* CST which dominated in the healthy control group. Since we could not analyze the healthy vaginal microbiota of those women that later developed BV, we cannot exclude that this CST dominated in our study cohort already before antibiotic treatment. However, it is more likely that treatment with metronidazole encouraged *L. iners* colonization more than other *Lactobacillus* species. It concurs with findings that *L. iners* and *L. crispatus* are negatively correlated in the vaginal microbiota [57]. The ambiguous role of *L. iners* has been widely discussed and it is believed that *L. crispatus* is protective for BV whereas *L. iners* may not be [5, 63]. *L. iners* adapts its expression profile in dysbiosis. Therefore, it has less stress than *L. crispatus* when it comes to fighting the colonization of BV-associated anaerobic species which makes *L. iners* colonization a predisposing factor for BV [64]. A study investigating women before and after acute BV may shed light into the question where the abundance of *L. iners* after metronidazole treatment originates.

The BV recurrence rate of 28% observed in our study group for microbiota analysis and about 20% regarding the clinical population is smaller than that found in another study, which was up to 60% over a period of 12 months [8]. Women were treated with pH-adapted pessaries (pH 4.5) containing either an amphoteric tenside or lactic acid, after metronidazole therapy, which could be an influencing factor. A placebo or an untreated control group was not included in the study and therefore could not be used for direct comparison. Since our study covered a period of 4 months, we may also be in line with published recurrence rates that rely on longer periods of time. It is noteworthy to mention that our study only included women with true BV infection. This was assured by strict inclusion criteria to select for the presence of biofilms. In other studies, weaker inclusion criteria might have led to inclusion of patients with transient or mild BV forms, when comparing recurrence rates this fact also should be considered. Nevertheless, our follow-up period ended approximately 3 months after metronidazole treatment. Because our data show an increase in microbial diversity and a decrease in *Lactobacillus* sp. colonization until visit 5, the recurrence rate within the following months is likely to have increased. Recurrence of BV occurred in woman harboring the *L. iners* CST as well as in those with the *L. crispatus* CST. Moreover, recurrence rates were equally high in women treated with LAP or WO 3191 pessary, and no particular species could be associated with recurrence. However, more women with higher microbial diversity at visit 3 developed recurrence than women with lower diversity, suggesting that a diverse CST can predispose to recurrence of BV. However, due to the complex etiology of BV many other parameters might play a role, e.g., host characteristics, *Lactobacillus* phages, sexual activity, or smoking [45].

Stabilizing the low diversity of the healthy vaginal microbiota seems to be the most promising approach in order to prevent recurrence of BV. This could be achieved by a combination of various measures, including dissolving the biofilm, stabilizing the vaginal pH, applying compounds that promote growth of *Lactobacillus* sp*.*, and providing live probiotics, e.g., strains of *L. crispatus*. For women that are colonized by an unstable but healthy vaginal community, this may prevent tipping the balance towards BV in response to external factors.

## Conclusions

This study indicates that *G. vaginalis* was not the main pathogen in bacterial vaginosis biofilms. After having been cured by the antibiotic metronidazole, recurrence was more likely in women that maintained a diverse microbiota. The pessary was unable to prevent recurrence. Thus, we suggest stabilizing the low diversity healthy flora by promoting growth of health-associated Lactobacillus sp. such as *L. crispatus.*

## Additional files

Additional file 1: FigureS1. Study overview, disposition of patients, visits performed and sampling for microbiota analysis (MB-A). The maximum study duration from visit 2 to visit 5 for each individual was 120 days. The initial treatment phase with metronidazole after visit 1 lasted 7–28 days. The follow-up examination at visit 5 took place 84 (+ 14) days after visit 4. 44 women were randomized and 43 treated. For safety analysis safety evaluable population was analyzed (*N* = 43; WO3191 = 18; LAP = 25). For evaluation of clinical efficacy full analysis set population (*N* = 37; WO3191 = 15; LAP = 22) and follow-up (*N* = 30; WO3191 = 11; LAP = 19) was used. *Discontinuation visit after visit 2 was assigned to visit 3. LAP = lactic acid pessaries. (TIFF 1224 kb)

Additional file 2: Table S1. List of raw and final sequencing results. (XLSX 2810 kb)

Additional file 3: Figure S2. A) Contaminants removed from the dataset. The most common OTUs and those identified as contaminants are shown for the cohort of healthy women in which they occurred. Contaminants are bold, regular members of the vaginal microbiota are shown transparent. B) A comparison of the reads for the two most abundant *Sneathia* sp. and the number of total reads clustered with a 97% and a 99% similarity threshold. (TIFF 2457 kb)

Additional file 4: Figure S3. A) Exclusion criteria and comparison of EPS, Nugent score, pH, age and biofilm of women with BV who were either included or excluded from the study. B) Frequency of global tolerability ratings (patients and investigators). C) Assessment of local tolerability by the intensity of solicited adverse device events. Individual mean values (mean_score_V3+V4_) and individual maximum values (max_score_V3+V4_) of investigator ratings for objective findings. (TIFF 1657 kb)

Additional file 5: Table S2. Change in “biofilm EPS*” status of patients between the visits. Number of patients profiting from treatment with either WO 3191 or LAP at visits 3 and 4 based on visit 2, and at visit 5 based on visit 4. The number of those patients is listed who went from positive/present to negative/absent biofilm, in these patients the “biofilm EPS*” status is improving. Also the number of patients is listed who were biofilm EPS negative beforehand and then changed being positive, which is an undesired outcome (worsening).The “net number of patients profiting” is calculated by the number of ‘improvements’ (change from biofilm EPS “positive” to “negative”) minus the number of ‘worsenings’ (change from biofilm EPS “negative” to “positive”). (DOCX 18 kb)

Additional file 6: Figure S4. Phylogenetic tree of the genus *Lactobacillus*. 16S rRNA gene sequences of all OTUs assigned to the genus *Lactobacillus* were aligned with reference sequences. (TIFF 115 kb)

Additional file 7: Figure S5. Rarefaction curves of all samples and resampling analysis. A) Samples were grouped according to health, inclusion/exclusion at visit 1 and visits 2–5. The x-axis was cut at the mean value of sequencing depth. B) Distribution of all samples with a sequencing depth above and below 5000 reads shown in a PCO. C) Resampling to the lowest sequencing depth was performed 20 times for 5 randomly chosen samples with low to high sequencing depth: (A) 1319 reads (B) 25,726 reads (C) 26,790 reads (D) 93,244 reads (E) 213,795 reads. The standard error (standard deviation of the mean) is indicated. (TIFF 601 kb)

Additional file 8: Figure S7. Biomarkers for BV. LEfSe biomarker analysis comparing the healthy cohort to the excluded and the included group of women with BV (LDA threshold = 2.0). (TIFF 3840 kb)

Additional file 9: Figure S6. Microbiota composition of all screened women with BV and of the healthy control group. A) Individual microbial profiles of all healthy women and women at visit 1 (included and excluded). Clustering of samples is based on Pearson correlations within each group. B) Individual microbial profiles of all included women at visit 2 to 5 according to treatment group. OTUs below 1% abundance are summarized as “others”. (TIFF 2160 kb)

Additional file 10: Figure S8. Analysis of recurrence. PCO and LEfSe analysis of (A) samples associated with positive or negative metronidazole treatment outcome at visit 1 before administration of metronidazole, (B) samples at visit 3 before BV recurrence at visit 4 and (C) samples at visit 4 before BV recurrence at visit 5. (TIFF 1060 kb)
