# Supplementary figures and images for: Treatment of biofilms in bacterial vaginosis by an amphoteric tenside pessary-clinical study and microbiota analysis
Source: Microbiome. 2017 Sep 13;5:119. doi: 10.1186/s40168-017-0326-y (PMC5598074; doi:10.1186/s40168-017-0326-y)

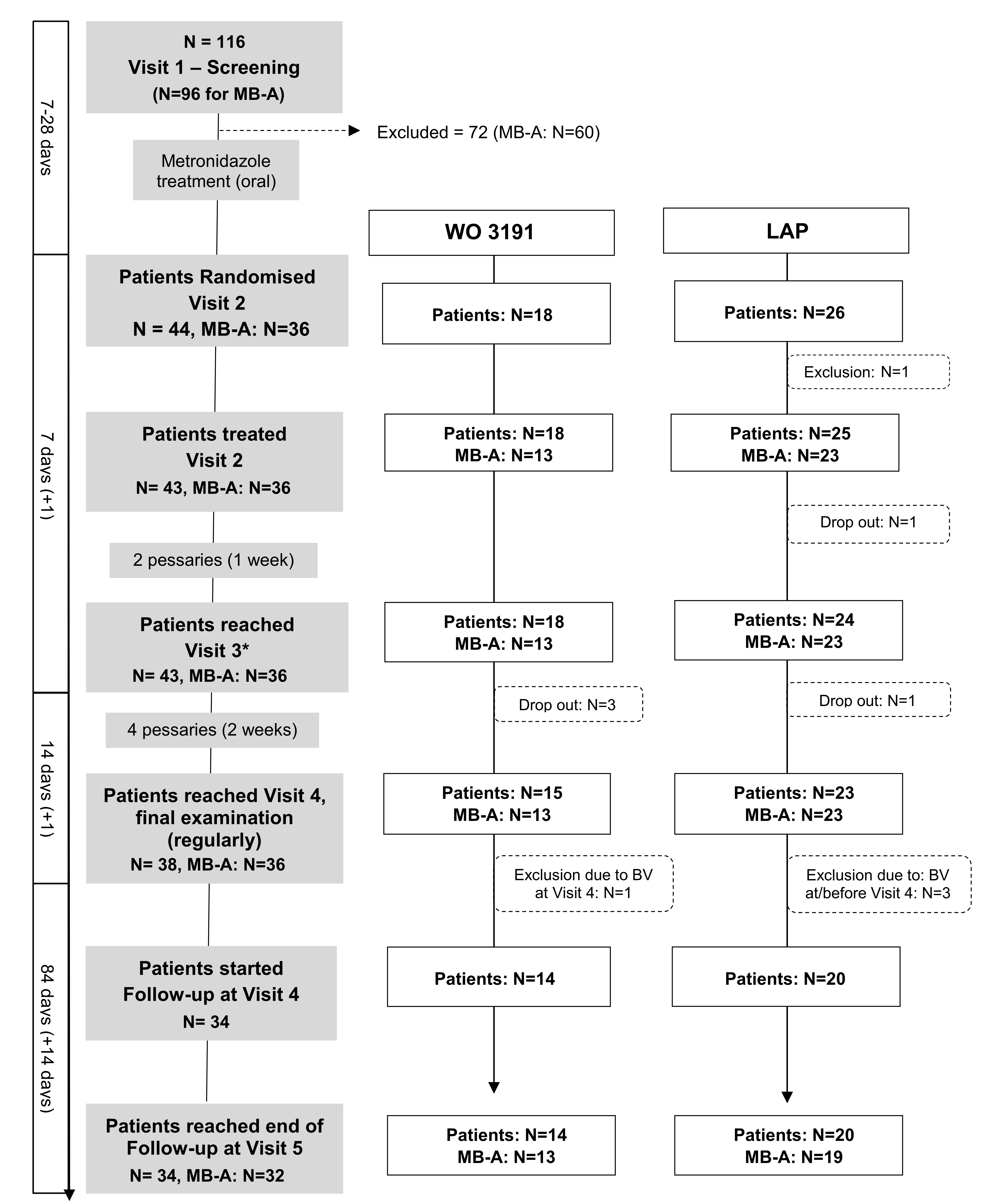

Supplement: Supplementary file 1 — Study overview, disposition of patients, visits performed and sampling for microbiota analysis (MB-A). The maximum study duration from visit 2 to visit 5 for each individual was 120 days. The initial treatment phase with metronidazole after visit 1 lasted 7–28 days. The follow-up examination at visit 5 took place 84 (+ 14) days after visit 4. 44 women were randomized and 43 treated. For safety analysis safety evaluable population was analyzed (N = 43; WO3191 = 18; LAP = 25). For evaluation of clinical efficacy full analysis set population (N = 37; WO3191 = 15; LAP = 22) and follow-up (N = 30; WO3191 = 11; LAP = 19) was used. *Discontinuation visit after visit 2 was assigned to visit 3. LAP = lactic acid pessaries. (TIFF 1224 kb) [file 40168_2017_326_MOESM1_ESM.tif]

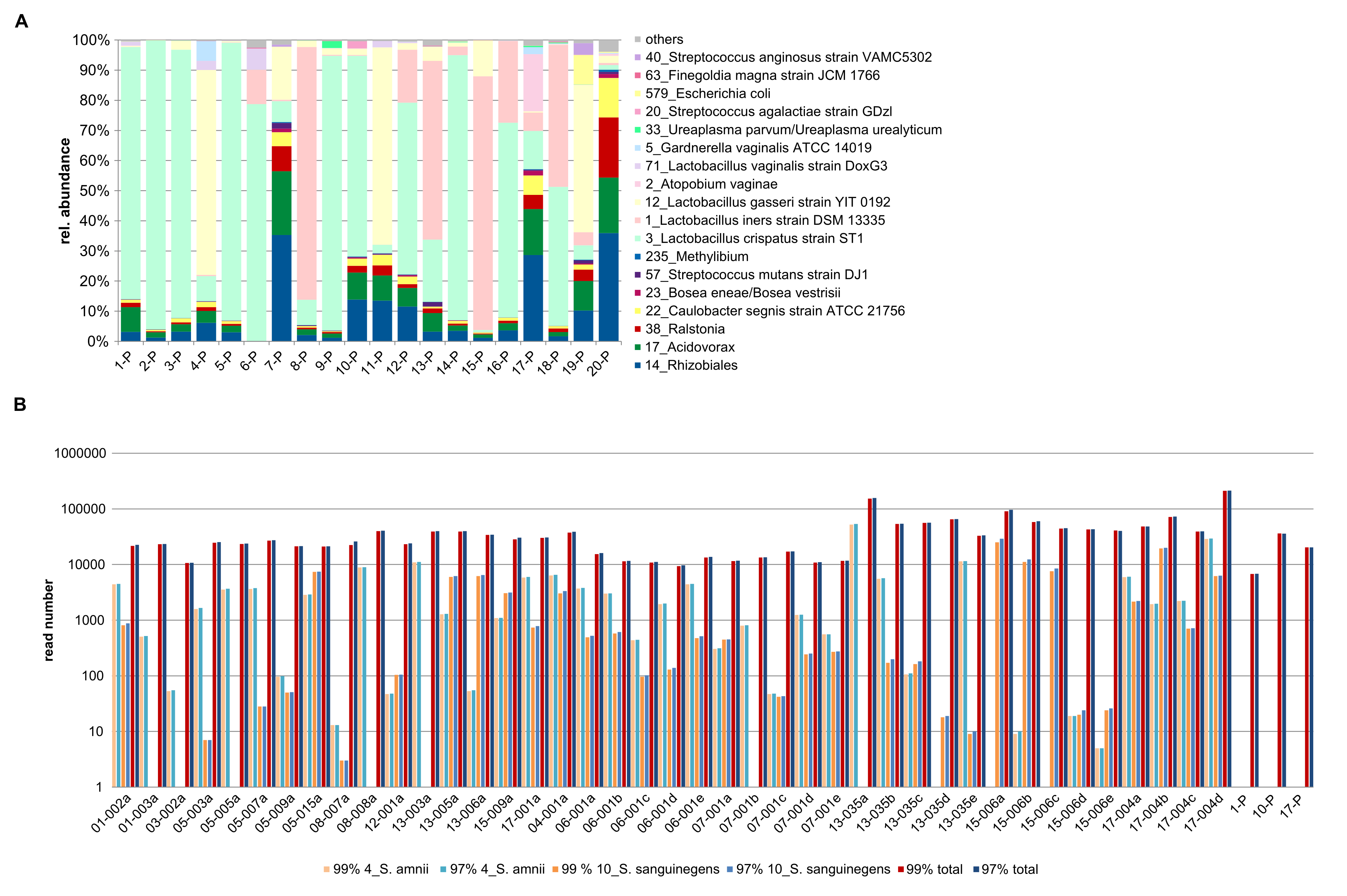

Supplement: Supplementary file 3 — A) Contaminants removed from the dataset. The most common OTUs and those identified as contaminants are shown for the cohort of healthy women in which they occurred. Contaminants are bold, regular members of the vaginal microbiota are shown transparent. B) A comparison of the reads for the two most abundant Sneathia sp. and the number of total reads clustered with a 97% and a 99% similarity threshold. (TIFF 2457 kb) [file 40168_2017_326_MOESM3_ESM.tif]

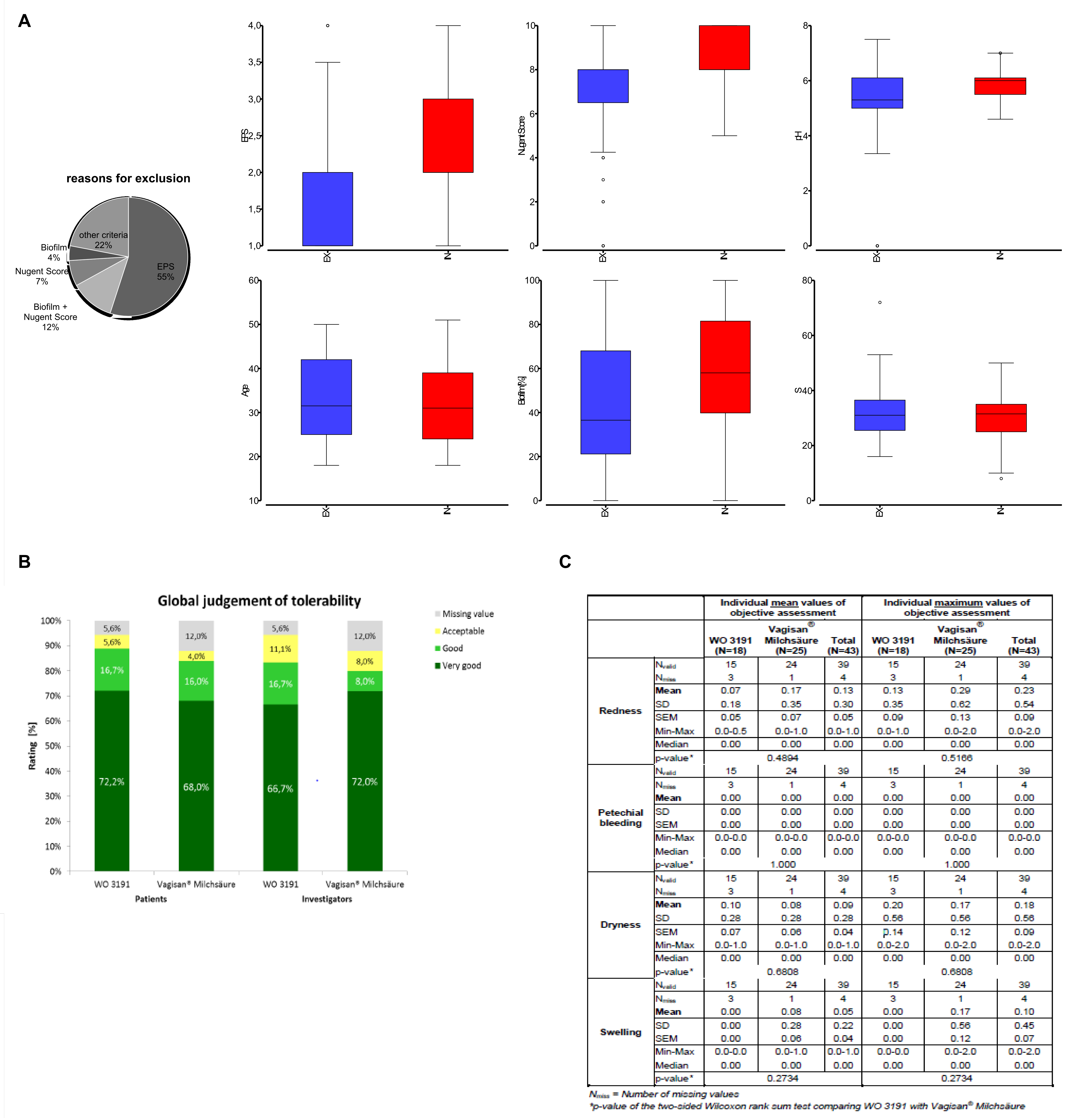

Supplement: Supplementary file 4 — A) Exclusion criteria and comparison of EPS, Nugent score, pH, age and biofilm of women with BV who were either included or excluded from the study. B) Frequency of global tolerability ratings (patients and investigators). C) Assessment of local tolerability by the intensity of solicited adverse device events. Individual mean values (meanscore_V3+V4) and individual maximum values (maxscore_V3+V4) of investigator ratings for objective findings. (TIFF 1657 kb) [file 40168_2017_326_MOESM4_ESM.tif]

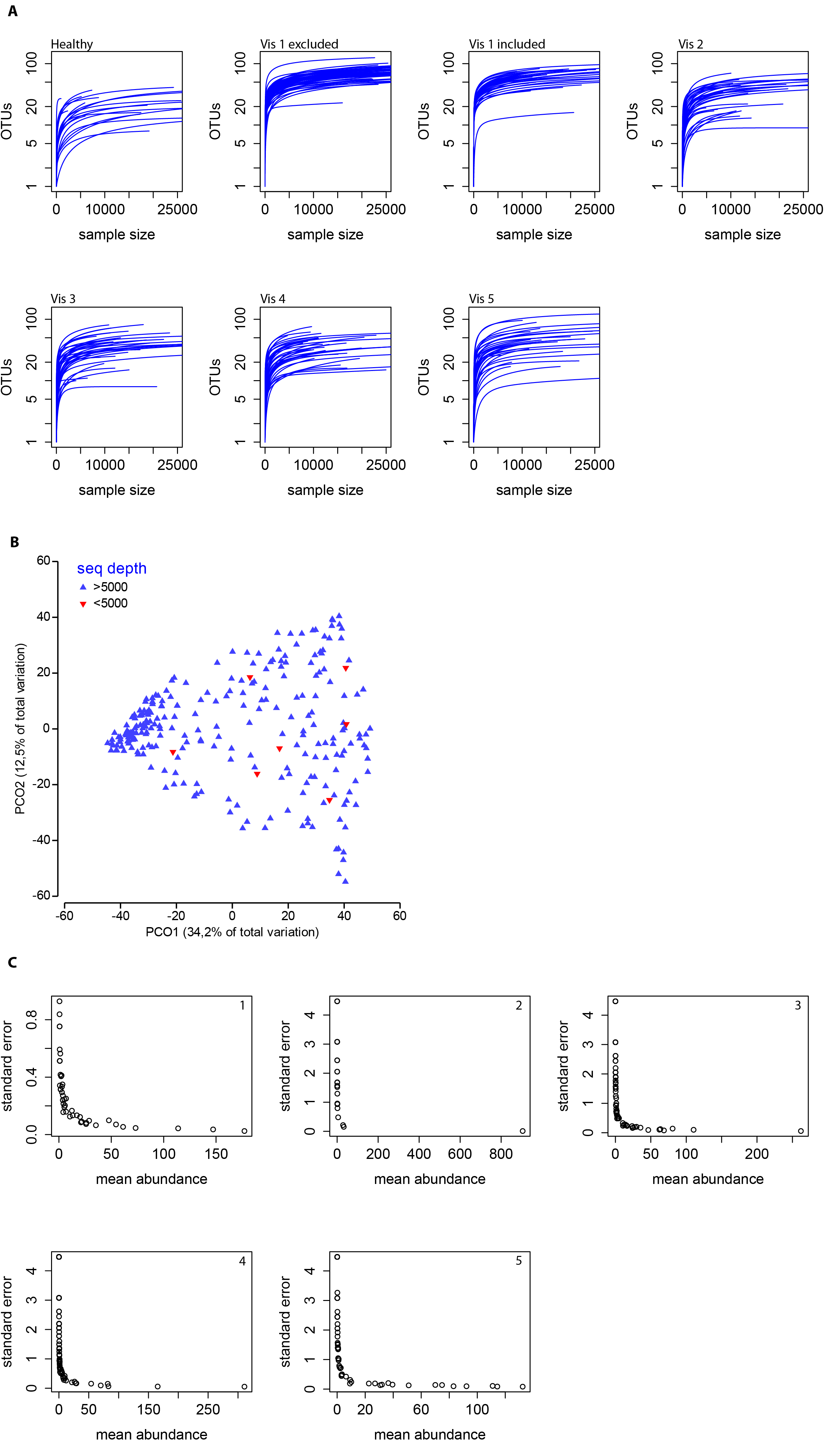

Supplement: Supplementary file 7 — Rarefaction curves of all samples and resampling analysis. A) Samples were grouped according to health, inclusion/exclusion at visit 1 and visits 2–5. The x-axis was cut at the mean value of sequencing depth. B) Distribution of all samples with a sequencing depth above and below 5000 reads shown in a PCO. C) Resampling to the lowest sequencing depth was performed 20 times for 5 randomly chosen samples with low to high sequencing depth: (A) 1319 reads (B) 25,726 reads (C) 26,790 reads (D) 93,244 reads (E) 213,795 reads. The standard error (standard deviation of the mean) is indicated. (TIFF 601 kb) [file 40168_2017_326_MOESM7_ESM.tif]

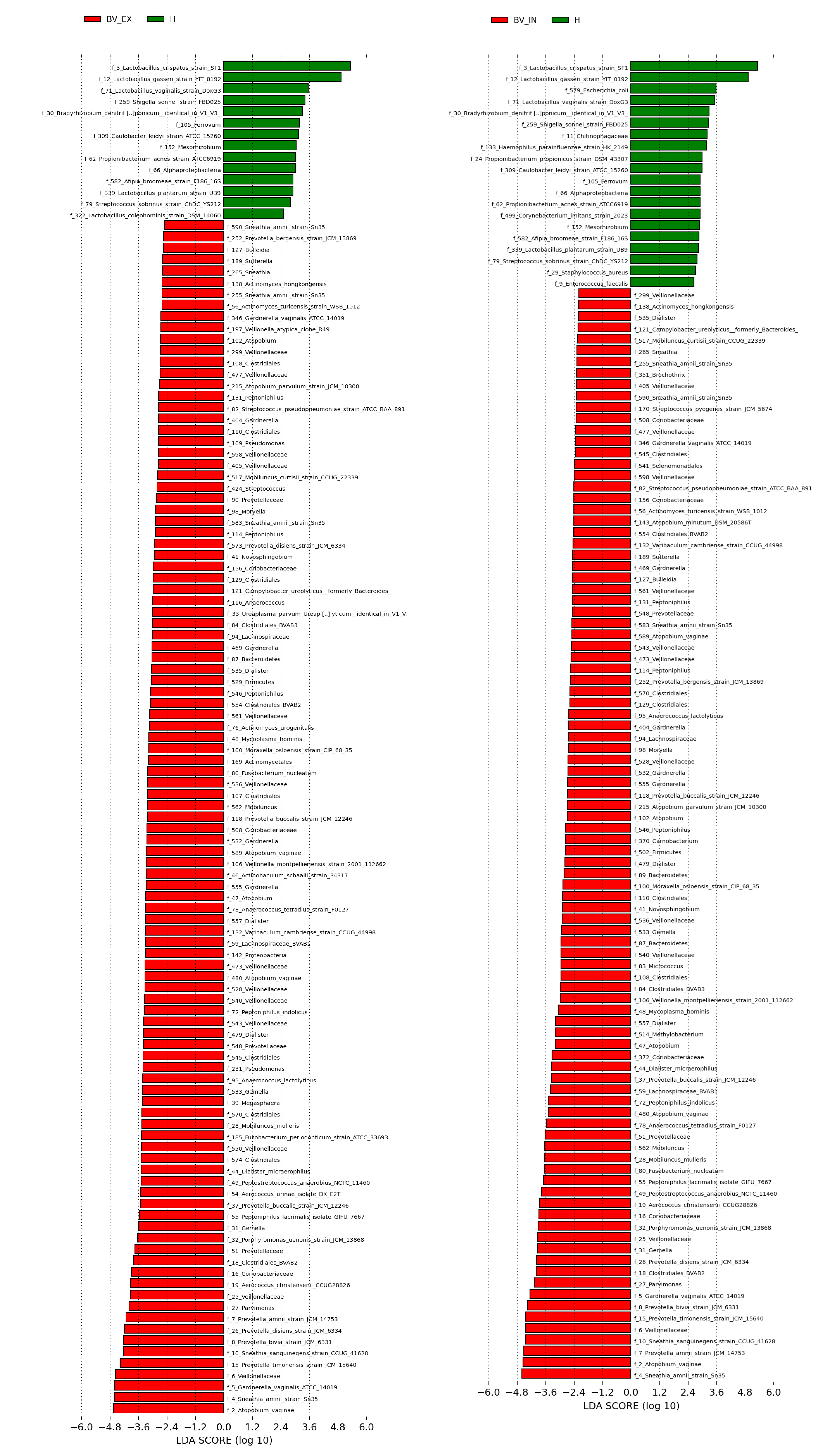

Supplement: Supplementary file 8 — Biomarkers for BV. LEfSe biomarker analysis comparing the healthy cohort to the excluded and the included group of women with BV (LDA threshold = 2.0). (TIFF 3840 kb) [file 40168_2017_326_MOESM8_ESM.tif]

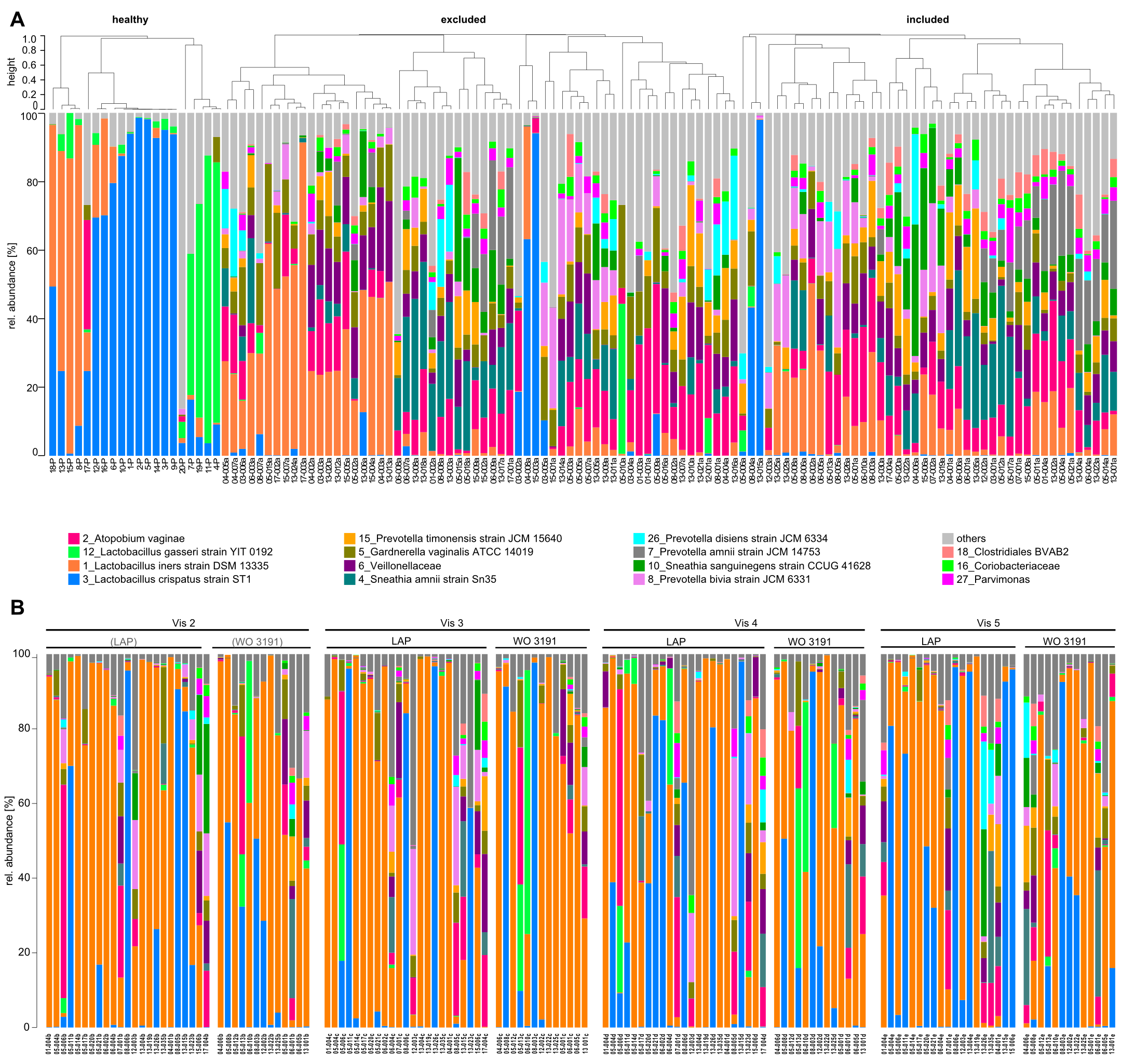

Supplement: Supplementary file 9 — Microbiota composition of all screened women with BV and of the healthy control group. A) Individual microbial profiles of all healthy women and women at visit 1 (included and excluded). Clustering of samples is based on Pearson correlations within each group. B) Individual microbial profiles of all included women at visit 2 to 5 according to treatment group. OTUs below 1% abundance are summarized as “others”. (TIFF 2160 kb) [file 40168_2017_326_MOESM9_ESM.tif]

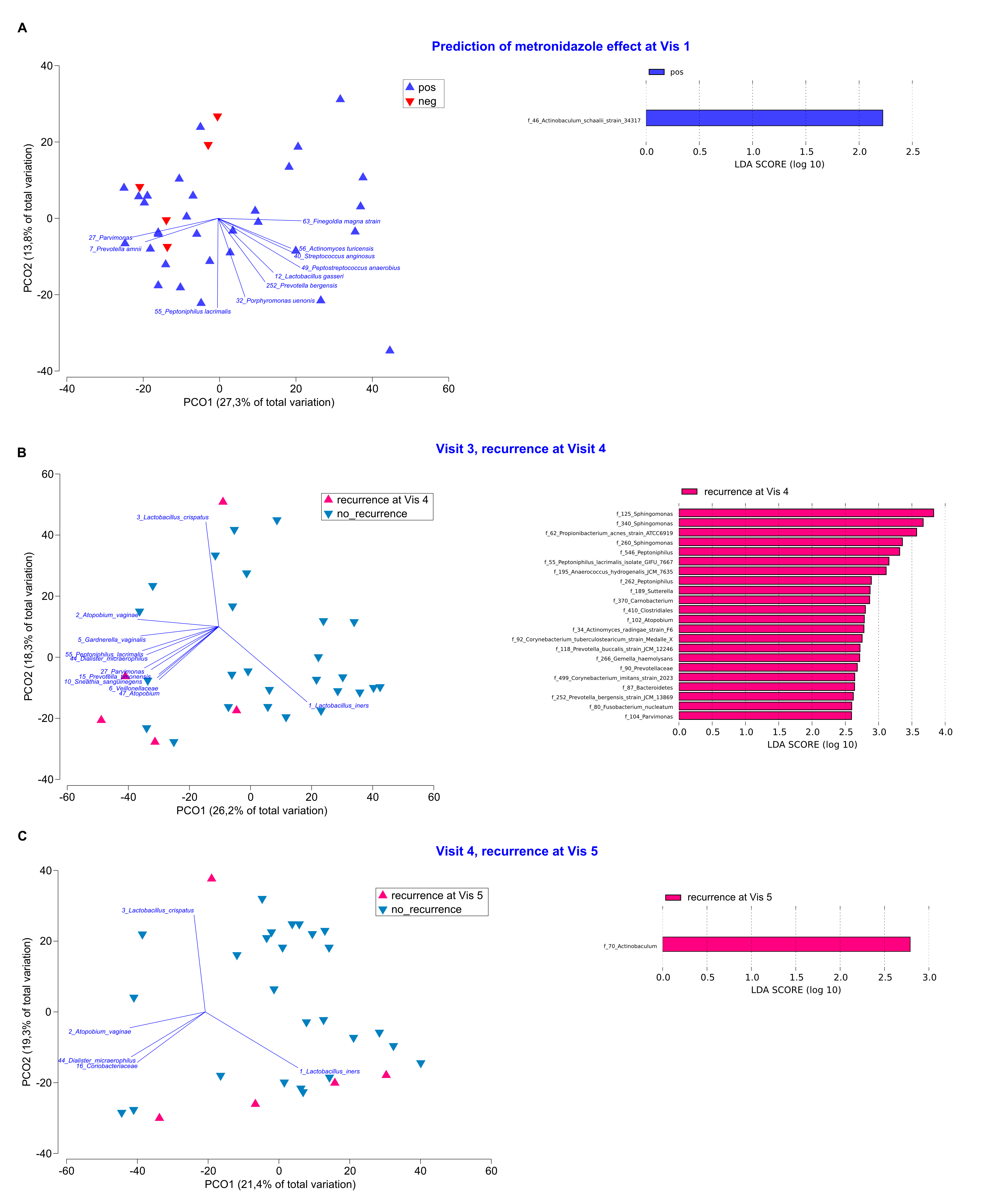

Supplement: Supplementary file 10 — Analysis of recurrence. PCO and LEfSe analysis of (A) samples associated with positive or negative metronidazole treatment outcome at visit 1 before administration of metronidazole, (B) samples at visit 3 before BV recurrence at visit 4 and (C) samples at visit 4 before BV recurrence at visit 5. (TIFF 1060 kb) [file 40168_2017_326_MOESM10_ESM.tif]
