# Supplementary material for: Treatment of biofilms in bacterial vaginosis by an amphoteric tenside pessary-clinical study and microbiota analysis
Source: Microbiome. 2017 Sep 13;5:119. doi: 10.1186/s40168-017-0326-y (PMC5598074; doi:10.1186/s40168-017-0326-y)
Supplement: Supplementary file 5 — Change in “biofilm EPS*” status of patients between the visits. Number of patients profiting from treatment with either WO 3191 or LAP at visits 3 and 4 based on visit 2, and at visit 5 based on visit 4. The number of those patients is listed who went from positive/present to negative/absent biofilm, in these patients the “biofilm EPS*” status is improving. Also the number of patients is listed who were biofilm EPS negative beforehand and then changed being positive, which is an undesired outcome (worsening).The “net number of patients profiting” is calculated by the number of ‘improvements’ (change from biofilm EPS “positive” to “negative”) minus the number of ‘worsenings’ (change from biofilm EPS “negative” to “positive”). (DOCX 18 kb) [file 40168_2017_326_MOESM5_ESM.docx]

**Table S2. Change in "biofilm EPS*" status of patients between the visits.** Number of patients profiting from treatment with either WO 3191 or LAP at visits 3 and 4 based on visit 2, and at visit 5 based on visit 4. The number of those patients is listed who went from positive/present to negative/absent biofilm, in these patients the "biofilm EPS*" status is improving. Also the number of patients is listed who were biofilm EPS negative beforehand and then changed being positive, which is an undesired outcome (worsening).The “net number of patients profiting” is calculated by the number of ‘improvements’ (change from biofilm EPS “positive” to “negative”) minus the number of ‘worsenings’ (change from biofilm EPS “negative” to “positive”).

| **Change in "biofilm EPS*" between the visits** | **Visit 2 to Visit 3** | | **Visit 2 to Visit 4***** | | **Visit 3 to Visit 4** | | **Visit 4 to Visit 5** | |
| --- | --- | --- | --- | --- | --- | --- | --- | --- |
| **(FAS)** | **WO 3191** | **LAP** | **WO 3191** | **LAP** | **WO 3191** | **LAP** | **WO 3191** | **LAP** |
| total number of patients | (N=15) | (N=22**) | (N=15) | (N=22**) | (N=15) | (N=22**) | (N=11) | (N=19) |
| **number of patients with improved status (present to not present)** | 2 of 6 | 3 of 6 | 5 of 6 | 1 of 5 | 3 of 4 | 1 of 3 | 1 of 1 | 3 of 5 |
| **Number of patients with worsened status (not present to present)** | 0 of 9 | 1 of 16 | 0 of 9 | 3 of 16 | 0 of 11 | 5 of 18 | 2 of 10 | 4 of 14 |
| number of patients with unchanged status | 13 of 15 | 18 of 22 | 10 of 15 | 17 of 22 | 12 of 15 | 16 of 22 | 10 of 15 | 14 of 22 |
| **Net number of patients profiting:** improved minus worsening | **2** | **2** | **5** | **-2** | **3** | **-4** | **-1** | **-1** |

* “Biofilm EPS” is the combined parameter of vaginal biofilm + EPS in urine. Extracellular polymeric substances (EPS) in urine were measured by enzyme-linked lectine assay, vaginal biofilms were detected by crystal violet staining. “Biofilm EPS” was considered positive/present if a vaginal biofilm AND EPS in urine were present. If only one parameter of the combined parameter was positive/present the “Biofilm EPS” was considered negative/not present.

** *One patient with missing value at Visit 3 and Visit 4 is not considered for improvement/worsening in this table*

*****Significant difference between the two treatment groups regarding the net number of patients profiting between Visit 2 and Visit 4 (p=0.047 , Fisher´s exact test, two sided, 95% confidence intervals).
